# Association of Vitamin D with Perfluorinated Alkyl Acids in Women with and without Non-Obese Polycystic Ovary Syndrome

**DOI:** 10.3390/biomedicines12061255

**Published:** 2024-06-05

**Authors:** Alexandra E. Butler, Thozhukat Sathyapalan, Priya Das, Edwina Brennan, Stephen L. Atkin

**Affiliations:** 1 Research Department, Royal College of Surgeons of Ireland, Busaiteen 15503, Bahrain; pdas@rcsi.com (P.D.); ebrennan@rcsi-mub.com (E.B.); satkin@rcsi.com (S.L.A.); 2 Academic Endocrinology, Diabetes and Metabolism, Hull York Medical School, Hull HU6 7RU, UK; thozhukat.sathyapalan@hyms.ac.uk

**Keywords:** endocrine disrupting chemicals, PFAAs, perfluorinated alkyl acids, PFOA, PFOS, PFNA, PFHxS, polycystic ovary syndrome, PCOS, IVF

## Abstract

Background. Perfluorinated alkyl acids (PFAAs) are persistent organic pollutants affected by BMI and ethnicity, with contradictory reports of association with vitamin D deficiency. Methods. Twenty-nine Caucasian women with non-obese polycystic ovary syndrome (PCOS) and age- and BMI-matched Caucasian control women (*n* = 30) were recruited. Paired serum samples were analyzed for PFAAs (*n* = 13) using high-performance liquid chromatography–tandem mass spectrometry. Tandem mass spectrometry determined levels of 25(OH)D_3_ and the active 1,25(OH)_2_D_3_. Results. Women with and without PCOS did not differ in age, weight, insulin resistance, or systemic inflammation (C-reactive protein did not differ), but the free androgen index was increased. Four PFAAs were detected in all serum samples: perfluorooctane sulfonic acid (PFOS), perfluorooctanoic acid (PFOA), perfluorononanoic acid (PFNA), and perfluorohexane sulfonic acid (PFHxS). Serum PFOS was higher in PCOS versus controls (geometric mean [GM] 3.9 vs. 3.1 ng/mL, *p* < 0.05). Linear regression modeling showed that elevated PFHxS had higher odds of a lower 25(OH)D_3_ (OR: 2.919, 95% CI 0.82–5.75, *p* = 0.04). Vitamin D did not differ between cohorts and did not correlate with any PFAAs, either alone or when the groups were combined. When vitamin D was stratified into sufficiency (>20 ng/mL) and deficiency (<20 ng/mL), no correlation with any PFAAs was seen. Conclusions. While the analyses and findings here are exploratory in light of relatively small recruitment numbers, when age, BMI, and insulin resistance are accounted for, the PFAAs do not appear to be related to 25(OH)D_3_ or the active 1,25(OH)_2_D_3_ in this Caucasian population, nor do they appear to be associated with vitamin D deficiency, suggesting that future studies must account for these factors in the analysis.

## 1. Introduction

Polycystic ovary syndrome (PCOS) is the most common endocrinological disorder in women of reproductive age, affecting 6–20% [1,2,3,4]. PCOS is associated with infertility, hirsutism, and acne, as well as metabolic dysregulation with the development of diabetes and an increased cardiovascular risk [5,6,7]. Studies have previously reported associations of perfluorinated alkyl acids (PFAAs) with menstrual irregularity and infertility [8,9], though those results may have been affected by the inclusion of women with PCOS. PFAAs have been linked to the development of PCOS [10], and others have reported that PCOS differs from controls for environmental pollutant levels, with PCOS subjects having increased perfluorooctanoic acid (PFOA) and perfluorooctane sulfonic acid (PFOS) levels [11].

PFAAs are synthetic chemicals that are widely used in surface protective coatings, surfactants, clothing and other textiles, and fire-fighting foams, of which PFOS and PFOA are the most abundant, and both are considered persistent organic pollutants [12]. PFAAs contain a fluorinated, hydrophobic alkyl chain and a hydrophilic end group. Human PFAA exposure is primarily caused by ingestion, inhalation, or direct contact, with dietary intake accounting for the most frequent exposure mechanism [13]. The consumption of eggs, meat, milk, and seafood is reported to be the most significant contributors to PFAA exposure in the human diet [14]. Such contamination of food stuff is the result of direct contact during production, processing, packaging, and storage, or through contamination of the soil, water, or air used in food production, consumption of food from animals that have been exposed through contaminated feed, environmental exposure, or accumulation in the aquatic food chain [15]. PFAAs are known to persist and accumulate, and their chain length determines their elimination; they particularly become sequestered in the kidney and liver, and their levels are BMI-dependent [16]. They are bound in a non-covalent manner to serum proteins, especially albumin [17,18], with an elimination half-life from serum of ~5.4 and 3.8 years for PFOA and PFOS, respectively [19,20]. PFOS and PFOA are toxic to the immune system [12], and PFAAs have both estrogenic and anti-estrogenic activities in vitro [21]. It is currently not known whether PFAAs may differentially affect the different PCOS phenotypes. The Rotterdam consensus [22] diagnostic criteria include clinical/biochemical hyperandrogenism, oligomenorrhea, amenorrhoea, and polycystic ovaries as assessed by transvaginal ultrasound (TVUS), thus giving four different PCOS phenotypes, A to D. PCOS phenotype A, which expresses all three of the diagnostic criteria, is reported to be at higher risk of adverse metabolic and cardiovascular outcomes compared to the other phenotypes, and phenotype D is the least severe [23].

Vitamin D_3_ (cholecalciferol) is manufactured endogenously in the skin through the action of ultraviolet B radiation (UV-B) upon 7-dehydrocholesterol, leading to its hydroxylation. 25-hydroxy vitamin D_3_ (25(OH)D_3_) is transported to the kidney, where it is converted by 1-alpha hydroxylase to 1,25(OH)_2_D_3_, the active form [24]. Macrophages also produce 1,25(OH)_2_D_3_ via the type 2 interferon response, leading to its extrarenal production [25]. Vitamin D deficiency is a global issue [26], and vitamin D insufficiency/deficiency has been strongly linked to a variety of negative health outcomes, examples being osteoporosis and an increased incidence of type 2 diabetes with poorer glycemic control, as well as cancer, cardiovascular disease, autoimmune diseases, and depression. In addition, an increase in mortality has been documented that can be circumvented with the use of vitamin D supplements [27,28]. Vitamin D has been reported to play a significant modulatory role in systemic inflammation and in the immune response, with its inherent anti-inflammatory effects inducing alterations in macrophage and T cell responses [25,29,30]. Deficiency of vitamin D is commonly found in PCOS, with 67–85% being severely deficient [31,32]. Vitamin D deficiency is associated with insulin resistance and obesity [33], both common conditions in PCOS, with vitamin D being sequestered in adipocytes. It has been suggested that vitamin D deficiency may then exacerbate the phenotypic features of PCOS [34] and has been shown to correlate to cardiovascular risk markers, though research has suggested that vitamin D deficiency does not exacerbate features of cardiovascular risk in PCOS [35]. Studies indicate that vitamin D supplementation (commonly given in the form of oral vitamin D_2_ supplements) is beneficial in countering insulin resistance and steroidogenesis of estradiol and progesterone in obese PCOS women [36,37]. A systematic review with meta-analysis suggested that vitamin D deficiency was associated with insulin resistance, but the significance was lost when BMI was statistically accounted for [33].

In a study of 7040 individuals, elevation in PFOS was associated with lower total vitamin D (25(OH)D_3_) levels, with a much stronger association noted amongst Caucasians and those individuals >60 years old [38]. Recent reports indicate that PFAA exposure is associated with cardiovascular disease, diabetes, and vitamin D deficiency, but studies in humans have provided contradictory results that require further evaluation of the biological toxicity of PFAAs [39]. Further studies have reported that, in pregnant African American women, PFAA exposure may affect vitamin D levels [40] and that perfluorohexane sulfonate (PFHxS) correlates strongly with vitamin D_3_ metabolites [41]. In silico, PFOA bound competitively to the Vitamin D Receptor (VDR), and, indeed, mixtures of PFAAs may also activate the VDR, which has a critical role in the regulation of genes important for the maintenance of calcium homeostasis and immune and endocrine functions [42].

It can, therefore, be seen from the above that PFAA exposure is associated with vitamin D deficiency, PCOS is associated with vitamin D deficiency, and PFAAs are associated with PCOS, but all of the studies have been undertaken in PCOS subjects where obesity may confound with increased insulin resistance and BMI; insulin resistance (IR) and obesity are tightly linked with PCOS, affect vitamin D status, and potentially also PFAA levels, and IR and obesity are not easy to account for statistically. The only way to address this issue directly is to have a study design of non-obese women with PCOS who are additionally non-insulin resistant to determine whether or not the inherent pathophysiology of PCOS with vitamin D is associated with PFAAs; therefore, we correlated vitamin D_3_ and 1,25(OH)_2_D_3_ (its active metabolite) with levels of PFAAs in non-obese, non-insulin-resistant PCOS women versus a matched control cohort with the hypothesis that, when these parameters were accounted for, there would be no difference between groups.

## 2. Materials and Methods

This prospective exploratory cohort study recruited women from the Hull IVF Unit, UK. Ethical approval was granted by the Yorkshire and the Humber NRES ethical committee, UK (approval number 02/03/043) [43]. The PCOS women were recruited according to the revised Rotterdam ESHRE/ASRM-sponsored PCOS consensus workshop group 2003 criteria; as such, PCOS was diagnosed if 2 of 3 criteria were present: menstrual disorder (oligo/amenorrhoea), clinical/biochemical hyperandrogenism, or ultrasound-determined polycystic ovaries [22]. Recruitment criteria necessitated an age between 20 and 45 years and a BMI ≤ 29. Exclusion criteria included liver or renal disease, diabetes, immune system disease, acute/chronic infections, or inflammatory disorders. A comparable study upon which to establish a formal power calculation was unavailable; when power and sample size for pilot studies were reviewed [44], the conclusion reached was that 20 degrees of freedom as a minimum was required for the estimation of variability and effect size. Thus, we undertook recruitment of 25 women per group plus 5 extra women as a buffer to allow for dropouts and covariate adjustment. In line with this, 59 women were recruited, half with PCOS (*n* = 29) and half without (*n* = 30), with control women being age- and BMI-matched (Table 1).

### 2.1. Sample Collection

Blood samples (venous, fasting) were drawn and centrifuged (3500× *g*, 15 min, 4 °C), and the aliquots were stored (−80 °C) <1 h post-collection. An insulin immunoassay was performed on serum using a competitive chemiluminescent method on a DPC Immulite 2000 analyzer (Euro/DPC, Llanberis, UK). The determination of C-reactive protein (CRP) was accomplished using an enzymatic method on a Synchron LX20 analyzer (Beckman-Coulter, High Wycombe, UK). Thyroid hormone and estradiol immunoassays were undertaken using an Abbott Architect i4000 analyzer (Abbott Diagnostics Division, Maidenhead, UK). Androstenedione and testosterone levels (serum) were determined using liquid chromatography–tandem mass spectrometry (LC/MS/MS; Acquity UPLC-Quattro Premier XE-MS, Waters, Manchester, UK). The immunoassay with fluorescence detection for sex hormone-binding globulin (SHBG) was performed using a DPC Immulite 2000 analyzer with an upper assay limit of 2.0 nmol/L. Ion-exchange chromatography was undertaken to determine glycosylated hemoglobin A1c (HbA1c). 25(OH)D_3_ and 1,25(OH)_2_D_3_ levels were measured using isotope-dilution liquid chromatography–tandem mass spectrometry (LC-MS/MS) [45].

### 2.2. Analysis for PFAAs

The following thirteen PFAAs were measured: PFOS, PFOA, PFHxS, perfluorononanoic acid (PFNA), perfluorodecanoic acid (PFDA), perfluoropentanoic acid (PFPeA), perfluoroundecanoic acid (PFUnDA), perfluoroheptanoic acid (PFHpA), perfluorobutanesulfonate (PFBS), perfluorobutanoic acid (PFBA), perfluorohexanoic acid (PFHxA), perfluorodecane sulfonate (PFDS), and perfluorododecanoic acid (PFDoDA) (Table 2), as has been described previously [43]. Serum (200 µL) was prepared for analysis via high-performance liquid chromatography–tandem mass spectrometry (HPLC-MS/MS) employing a Nexera HPLC (Shimadzu Corp., Kyoto, Japan) paired with an API5500 QTRAP mass spectrometer (Sciex, Melbourne, Australia) with an electrospray ionization (ESI) interface operating in negative mode. Chromatographic separation was accomplished using a Gemini C_18_ column (50 × 2.0 mm, 4 µm; Phenomenex, Torrance, CA, USA), at 45 °C, a flow rate of 0.3 mL/min, and an injection volume of 5 µL. An isolator column (Phenomenex) was placed inline immediately after the mobile phase mixing chamber to delay the elution of solvent-derived background PFAA contamination. Data acquisition/processing was performed with Analyst^®^ TF 1.6 and MultiQuantTM software (version 2.0, Sciex) [43].

To calculate the estimated glomerular filtration rate (eGFR), the Modification of Diet in Renal Disease (MDRD) study method was utilized [46]. To calculate insulin resistance (IR), the homeostasis model assessment (HOMA) was employed using basal glucose and insulin levels ((Insulin × glucose)/22.5) [47]. The free androgen index (FAI) was determined by the following calculation: 100 × the serum testosterone:SHBG ratio. To determine the sum PFAA (∑PFAA), the molar concentrations of the four frequently detected PFAA compounds (PFOS, PFOA, PFHxS, and PFNA) were added together.

### 2.3. Statistics

This was an exploratory study, as there were no studies in the literature on which a power calculation could be performed. For the descriptive data, mean ± standard deviation (SD) was used for continuous data, while *n* (%) was used for categorical data. Student *t*-tests were undertaken for comparison of means as and when appropriate. Serum levels of PFAAs, hormones, and metabolic measures were evaluated for normality. Statistical significance was assumed at a *p*-value < 0.05, with the exception of exploratory Pearson correlation coefficient evaluations. Linear regression analyses using enter and backward elimination methods were used to model the changes in the continuous scores. Stata (IC 12.1, Stata Corp., College Station, TX, USA) was used to perform statistical analyses.

## 3. Results

Of the 29 PCOS subjects, 15 were classified as phenotype B and 14 as phenotype C, with none being phenotype A or C. Non-obese PCOS and controls were, according to the study design, matched for age and BMI and had similar indices of insulin resistance (HOMA-IR), CRP (a systemic inflammation marker), glycemia parameters (HbA1c), and thyroid function tests (thyroid stimulating hormone, triiodothyronine, and thyroxine) (Table 1). PCOS women had elevated FAI levels compared to controls. PCOS women also had a reduced mean eGFR versus the controls (88.3 vs. 97.4 mL/min/1.73 m^−2^, *p* < 0.05). 25(OH)D_3_ and 1,25(OH)_2_D_3_ were similar between cohorts (Table 1).

Descriptive statistics and detection frequencies for serum PFAAs are shown in Table 2, including the detection frequencies, geometric means, range of values, and lower limit of reporting [43]. Only four PFAAs could be detected in all samples, specifically PFOS, PFOA, PFNA, and PFHxS. Generally, PFOS was found to have the highest concentration, followed by PFOA, PFHxS, and PFNA. Geometric mean PFOS serum levels were elevated in women with PCOS (Table 1), while the other PFAAs did not differ between women with and without PCOS.

**Figure 1:**
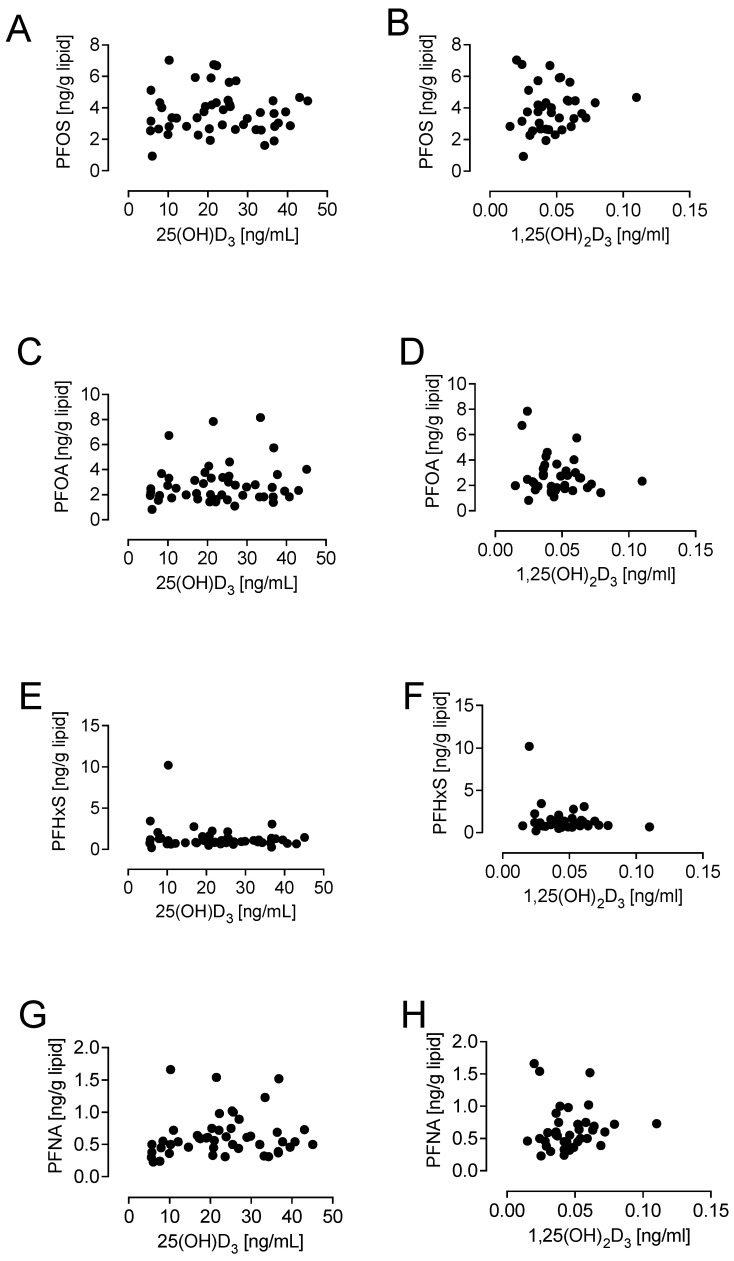
Lack of correlation of PFAAs with 25(OH)D_3_ and 1,25(OH)_2_D_3_ in combined groups of PCOS and control women. None of the PFAAs correlated with either 25(OH)D_3_ or 1,25(OH)_2_D_3_: PFOS (**A**,**B**), PFOA (**C**,**D**), PFHxS (**E**,**F**), and PFNA (**G**,**H**).

**Figure 2:**
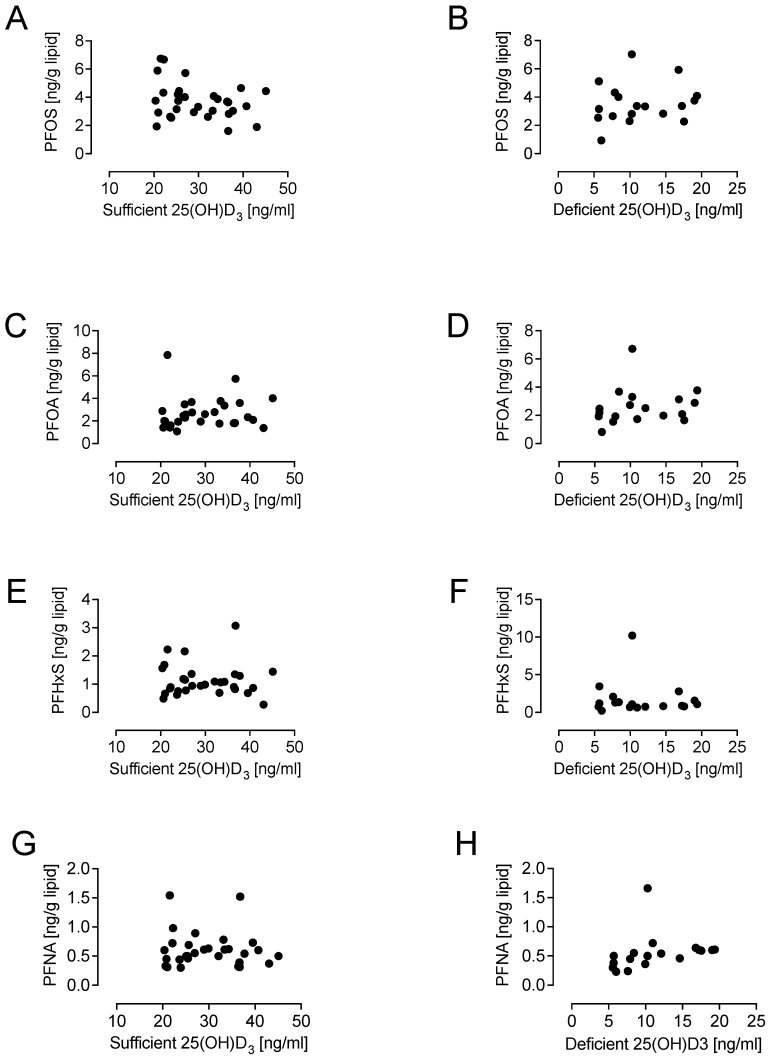
Lack of correlation of PFAAs with sufficient and deficient 25(OH)D3 in combined groups of PCOS and control women. None of the PFAAs correlated with either sufficient or deficient 25(OH)D3: PFOS (**A**,**B**), PFOA (**C**,**D**), PFHxS (**E**,**F**), and PFNA (**G**,**H**).

When PFOS, PFOA, PFHxS, and PFNA were separately correlated by cohort to 25(OH)D_3_, no significant correlation was found; therefore, the cohorts were combined to increase the power but still did not show any correlation between PFAAs and either 25(OH)D_3_ or 1,25(OH)_2_D_3_. It can be seen in Figure 1 that none of the PFAAs correlated with either 25(OH)D_3_ or 1,25(OH)_2_D_3_: PFOS (Figure 1A,B), PFOA (Figure 1C,D), PFHxS (Figure 1E,F), and PFNA (Figure 1G,H). When PFOS, PFOA, PFNA, and PFHxS were correlated as a combined group for vitamin sufficiency (>20 ng/mL) and vitamin D deficiency (<20 ng/mL) [41], still no correlation with PFOS, PFOA, PFHxS, or PFNA was found. It can be seen in Figure 2 that none of the PFAAs correlated with either sufficient or deficient 25(OH)D_3_: PFOS (Figure 2A,B), PFOA (Figure 2C,D), PFHxS (Figure 2E,F), and PFNA (Figure 2G,H).

A linear regression analysis was conducted to model the levels of 25(OH)D_3_ in response to the levels of the pollutants. Although the model was weak with an R^2^ of 0.116, it was noted that subjects with elevated levels of PFHxS had higher odds of having a lower level of 25(OH)D_3_ (OR: 2.919, 95% CI 0.82–5.75, *p* = 0.04).

## 4. Discussion

From the literature, it is recognized that PFAA exposure is associated with vitamin D deficiency, PCOS is associated with vitamin D deficiency, and PFAAs are associated with PCOS, but all the studies to date have been undertaken in PCOS subjects where obesity was a factor. In this study, the four detected PFAAs were consistent with previous reports [48,49]. The PCOS women had elevated geometric mean concentrations of PFOS versus controls, though concentrations of other PFAAs were similar between cohorts. While the linear regression model was weak, those subjects with higher PFHxS showed higher odds of having a lower 25(OH)D_3_. Others have reported that PCOS differs from controls for environmental pollutant levels, with PCOS subjects having higher perfluorooctanoic acid (PFOA) and perfluorooctane sulfonic acid (PFOS) levels [11]; however, in those studies, BMI, age, and the hormonal parameters of insulin resistance were not matched between participants. It has been noted that PFAA levels may be dependent on BMI [16], and this was accounted for in this study. Vitamin D may also be affected by obesity as it may be sequestered in adipose tissue [33], but, again, this was accounted for as the subjects were not obese. In the NHANES study of 7040 individuals, the increase in PFOS was associated with lower vitamin D concentrations, but this was also noted to be associated with whites and those >60 years of age; therefore, a mixed ethnic population may bias the results, and this was accounted for in this study by having only Caucasian white females within a narrow demographic area. Furthermore, the women with and without PCOS were on average around 30 years of age, with no difference between the groups that may have been associated with higher PFAA levels. While the PCOS women were not obese and did not differ from the control subjects, it is recognized that PCOS women have increased visceral fat mass compared to controls for any given weight and in both obese and non-obese PCOS [50]. While body morphology measurements were not performed (other than waist measurements that were not different between the groups), it may be surmised that the PCOS subjects may have had more visceral fat, which may have been responsible for the higher PFAAs recorded.

PFHxS is reported to correlate strongly with vitamin D_3_ metabolites [26] and PFOS with 1,25(OH)_2_D_3_ in women [51]. The linear regression results here with the higher PFHxS and lower odds of 25(OH)D_3_ would be in accord with the data presented by Etzel [38]. The mechanistic relationship between PFAAs and vitamin D is not fully understood. PFAAs are reported to interact with VDR. For example, in silico, PFOA is reported to compete with 1,25(OH)_2_D_3_ for the same binding site on VDR, and in vitro analysis in human osteoblasts revealed altered VDR target gene responsiveness and mineralization [52]. More recently, 14 PFAAs containing side-chain aromatics with steroid-backbone and extended aliphatic fluorocarbon sidechains were identified in silico to interact strongly with VDR, similar to natural ligands of VDR, including 1,25(OH)_2_D_3_, and more potently than that previously reported for PFOA [42]. Several of the identified PFAAs, including PFDoDA, were of regulatory concern given their frequent detection in human blood [42]. In serum, PFOS levels were associated with decreased lumbar bone mineral density in a cohort of premenopausal women (0.022 g/cm^2^ (95% CI: −0.038, −0.007; *p* = 0.006) [53], but whether PFAA VDR interaction is the cause in this cohort is unknown. Therefore, evidence of PFAA VDR interaction is sparse and limited to in silico and in vitro analyses, and further mechanistic studies in humans are required. In this study, none of the four PFAAs detected (PFOS, PFAS, PFHxS, or PFNA) correlated with either 25(OH)D_3_ or the active 1,25(OH)_2_D_3_. When the cohort was stratified according to vitamin D sufficiency and vitamin D deficiency, none of the four PFAAs (PFOS, PFAS, PFHxS, or PFNA) correlated with vitamin D. This suggests that additional factors as epiphenomena of vitamin D deficiency, such as obesity, insulin resistance, and ethnicity, are correlating with PFAAs rather than vitamin D per se. This would also address the comment that studies in humans have provided contradictory results that require further evaluation [39] and that prospective studies need to account for the parameters of BMI, insulin resistance, age, and ethnicity to definitively answer the question as to whether PFAAs are associated with vitamin D metabolism.

Renal elimination is a pathway for PFAA compound elimination [54,55]. Consequently, a reduced eGFR in PCOS women may provoke elevated serum PFAA concentrations for the same level of external exposure, and this has been observed with PFOS; however, this is not consistent with the finding that the other three PFAAs (PFAS, PFHxS, or PFNA) were not elevated, which may have resulted if a reduced eGFR accounted for PFOS. It has been suggested that eGFR may be lower in PCOS women than controls as a consequence of inflammation and is evidenced by an elevated CRP [56]; however, in this study, CRP was not different between cohorts. Therefore, most likely, the difference in eGFR was statistically, but not biologically, significant and may be accounted for by the error of measurement at this level of eGFR, where the error is still more than ±30 mL/min/1.73 m^2^ at 90 mL/min/1.73 m^2^ [57].

How vitamin D and PFAAs relate to the PCOS phenotype is unclear. Vitamin D receptor gene polymorphisms were associated with an increased risk of PCOS [58], suggesting that vitamin D levels may vary according to the PCOS phenotype. However, in a study of serum vitamin D levels in PCOS phenotypes A, B, and C, while vitamin D deficiency occurred more frequently in phenotype A, this was attenuated after adjusting for BMI and ethnicity; this suggests that there were no differences between the phenotypes once BMI was addressed [59], in accord with other reports [60]. There are no studies that have looked at PFAAs with PCOS phenotype, and, therefore, it is unknown whether there will be a differential effect of PFAAs or a difference in their levels in the different phenotypes; however, in light of this study with non-obese, non-insulin-resistant PCOS women with no systemic inflammation (normal CRP levels), it would seem unlikely that PFAAs would differ among PCOS phenotypes, though a larger robust cohort of PCOS phenotypes is necessary to prove this.

A strength, and a weakness, of this study was that all of the participants were Caucasian, removing any ethnic bias, and all were matched for age and BMI and were not insulin resistant or with systemic inflammation. However, when these confounders are accounted for, then there is the issue that much smaller differences may not have been seen due to the relatively small population under study. In addition, there are differing phenotypes of PCOS, with phenotype A being the most metabolic [23], and it is not inconceivable that the PFAA concentrations may relate to the different phenotypes given the association of PFAA with PCOS [10], but there were too few PCOS in phenotypes B and C to perform further sub-analysis.

In conclusion, while the analyses and findings here should be considered exploratory due to the relatively small sample sizes when age, BMI, and insulin resistance are accounted for, the PFAAs do not appear to be related to levels of 25(OH)D_3_ or its active form, 1,25(OH)_2_D_3_, in this Caucasian population, nor do they appear to be associated with vitamin D deficiency, suggesting that future studies must account for these factors in the analysis. However, PFOS was elevated in these non-obese PCOS women, perhaps due to increased visceral fat. Therefore, it may be that the relationship of PFAAs with vitamin D deficiency and PCOS is through the common theme of obesity with associated insulin resistance.

## Figures and Tables

**Table 1:** Demographics, hormones, laboratory reference ranges, biochemistry endpoints, and serum perfluorinated alkyl acid concentrations for PCOS patients and controls.

	Control (*n* = 30)	PCOS (*n* = 29)
	Mean ± SD	Mean ± SD
Age (years)	32.9 ± 4.6	30.7 ± 4.6
Body mass index (kg/m^2^)	25.6 ± 2.7	25.9 ± 2.8
Insulin (<10 μIU/mL)	7.9 ± 4.1 (*n* = 30)	7.9 ± 4.6 (*n* = 29)
HbA1C (<48 mmol/mol)	30.9 ± 6.5 (*n* = 27)	31.8 ± 3.0 (*n* = 28)
HOMA-IR	1.8 ± 1.0 (*n* = 30)	1.9 ± 1.6 (*n* = 29)
SHBG (nmol/L) (35–100 mmol/L)	104.2 ± 80.3 (*n* = 28)	71.7 ± 62.2 (*n* = 28)
Testosterone (nmol/L) (0–1.9 nmol/L)	0.85 ± 0.56 (*n* = 30)	1.04 ± 0.37 (*n* = 29)
Free androgen index (FAI)	1.44 ± 1.47 (*n* = 30)	3.32 ± 4.08 (*n* = 29) *
TSH (mU/L) (0.35–4.7 mU/l)	2.3 ± 1.0 (*n* = 27)	2.0 (0.8) (*n* = 28)
Free T3 (3.8–6 pmol/L)	4.8 ± 0.7 (*n* = 26)	4.8 ± 0.7 (*n* = 24)
Free T4 (7.8–21 pmol/L)	11.2 ± 1.3 (*n* = 26)	11.4 ± 2.2 (*n* = 24)
eGFR (>90 mL/min 1.73 m^−2^)	97.4 ± 18.9 (*n* = 28)	88.3 ± 10.1 (*n* = 28) *
C-Reactive Protein (0–8 mg L^−1^)	2.34 ± 2.34 (*n* = 27)	2.77 ± 2.57 (*n* = 28)
25(OH)D_3_ (20–40 ng/mL)	22.8 ± 11.5 (*n* = 30)	23.2 ± 10.9 (*n* = 29)
1,25(OH)_2_D_3_ (0.02–0.08 ng/mL)	0.05 ± 0.02 (*n* = 30)	0.05 ± 0.02 (*n* = 29)
Serum ^a^		
PFOS (ng/mL)	3.1 (2.6–3.6) (*n* = 30)	3.9 (3.4–4.4) (*n* = 29) *
PFOA (ng/mL)	2.4 (1.9–2.9) (*n* = 30)	2.4 (2.0–2.9) (*n* = 29)
PFHxS (ng/mL)	0.9 (0.8–1.2) (*n* = 30)	1.1 (0.9–1.4) (*n* = 29)
PFNA (ng/mL)	0.5 (0.4–0.6) (*n* = 30)	0.6 (0.5–0.7) (*n* = 29)

HbA1c = glycated hemoglobin; HOMA-IR = homeostatic model assessment for insulin resistance; SHBG = sex hormone-binding globulin; TSH = thyroid stimulating hormone; T3 = triiodothyronine; T4 = thyroxine; eGFR = estimated glomerular filtration rate; 25(OH)D_3_ = 25 hydroxy vitamin D3; 1,25(OH)_2_D_3_ = 1,25 dihydroxy vitamin D3; PFOS, perfluorooctane sulfonate; PFOA, perfluorooctanoic acid; PFHxS, perfluorohexane sulfonate; PFNA, perfluorononanoic acid; (the following perfluorinated alkyl acids were measured but not detected, as shown in Table 2: PFDA, perfluorodecanoic acid; PFPeA, perfluoropentanoic acid; PFUnDA, perfluoroundecanoic acid; PFHpA, perfluoroheptanoic acid; PFBS, perfluorobutanesulfonate; PFBA, perfluorobutanoic acid; PFHxA, perfluorohexanoic acid; PFDS, perfluorodecane sulfonate; and PFDoDA, perfluorododecanoic acid.). * *p* < 0.05; ^a^ geometric mean (95% CI).

**Table 2:** Summary of PFAA serum concentrations in PCOS case–control study (ng/mL).

	IUPAC Name	Detection Frequency *n* (%)	Geometric Mean (ng/mL)	Range (ng/mL)	LOR (ng/mL)
PFOS	1,1,2,2,3,3,4,4,5,5,6,6,7,7,8,8,8-heptadecafluorooctane-1-sulfonic acid	59 (100)	3.46	0.93–7.71	0.5
PFOA	2,2,3,3,4,4,5,5,6,6,7,7,8,8,8-pentadecafluorooctanoic acid	59 (100)	2.39	0.5–8.16	0.1
PFHxS	1,1,2,2,3,3,4,4,5,5,6,6,6-tridecafluorohexane-1-sulfonic acid	59 (100)	1.04	0.2–10.2	0.05
PFNA	2,2,3,3,4,4,5,5,6,6,7,7,8,8,9,9,9-heptadecafluorononanoic acid	59 (100)	0.57	0.2–1.79	0.2
PFDA	2,2,3,3,4,4,5,5,6,6,7,7,8,8,9,9,10,10,10-nonadecafluorodecanoic acid	45 (76)	0.31	<LOR-1.17	0.2
PFPeA	2,2,3,3,4,4,5,5,5-nonafluoropentanoic acid	29 (49)	-	<LOR-2.02	0.5
PFUnDA	2,2,3,3,4,4,5,5,6,6,7,7,8,8,9,9,10,10,11,11,11-henicosafluoroundecanoic acid	21 (36)	-	<LOR-0.47	0.2
PFHpA	2,2,3,3,4,4,5,5,6,6,7,7,7-tridecafluoroheptanoic acid	10 (17)	-	<LOR-0.70	0.1
PFBS	1,1,2,2,3,3,4,4,4-nonafluorobutane-1-sulfonate	4 (6.8)	-	<LOR-0.46	0.2
PFBA	2,2,3,3,4,4,4-heptafluorobutanoic acid	0	-	-	0.5
PFHxA	2,2,3,3,4,4,5,5,6,6,6-undecafluorohexanoic acid	0	-	-	0.5
PFDS	1,1,2,2,3,3,4,4,5,5,6,6,7,7,8,8,9,9,10,10,10-henicosafluorodecane-1-sulfonic acid	0	-	-	0.5
PFDoDA	2,2,3,3,4,4,5,5,6,6,7,7,8,8,9,9,10,10,11,11,12,12,12-tricosafluorododecanoic acid	0	-	-	0.5

The detection frequency (and percentage) was for the whole group (PCOS and controls) of 59 subjects, together with the geometric mean, range of values, and the lower limit of reporting (LOR); PFOS, perfluorooctane sulfonate; PFOA, perfluorooctanoic acid; PFHxS, perfluorohexane sulfonate; PFNA, perfluorononanoic acid; PFDA, perfluorodecanoic acid; PFPeA, perfluoropentanoic acid; PFUnDA, perfluoroundecanoic acid; PFHpA, perfluoroheptanoic acid; PFBS, perfluorobutanesulfonate; PFBA, perfluorobutanoic acid; PFHxA, perfluorohexanoic acid; PFDS, perfluorodecane sulfonate; PFDoDA, perfluorododecanoic acid.

## Data Availability

Data will be made available upon reasonable request to the corresponding author.
